# Lumican overexpression exacerbates lipopolysaccharide-induced renal injury in mice

**DOI:** 10.3892/mmr.2015.3940

**Published:** 2015-06-16

**Authors:** XIAO-MEI LU, LING MA, YU-NAN JIN, YAN-QIU YU

**Affiliations:** Department of Pathophysiology, College of Basic Medical Sciences, China Medical University, Shenyang, Liaoning 110001, P.R. China

**Keywords:** lipopolysaccharide, acute renal failure, apoptosis

## Abstract

The present study aimed to investigate the role of lumican in mice with endotoxin-induced acute renal failure (ARF). Lumican transgenic mice and wild-type mice were injected with lipopolysaccharide (LPS; 10 mg/kg) to establish a model of ARF. The mice were sacrificed at 24 h and the blood and renal tissue samples were collected. The value of serum creatinine (SCr) and blood urea nitrogen (BUN) were measured to determine renal function. An ELISA was used to determined the concentrations of renal cytokines, including tumor necrosis factor (TNF)α, interleukin (IL)-6, IL-4 and IL-10. The protein expression levels of Toll-like receptor (TLR4) and nuclear factor (NF)κB in renal tissues were assessed using western blot analysis. Terminal deoxynucleotidyl transferase-mediated dUTP nick end labeling was performed to monitor apoptosis of renal tissue. Light microscopy and electron microscopy were used to observe structural changes in the renal tissues. Following the administration of LPS, the SCr and BUN values of mice in the lumican transgenic group were higher compared with those in the control group. The expression levels of renal TLR4, NFκB, TNFα, IL-6, IL-4 and IL-10 were upregulated in the lumican transgenic mice compared with those in the wild-type control group. Apoptosis was detected predominantly on the renal tubule. There was a significant difference in the optical density of apoptotic bodies between the control mice and the lumican transgenic mice. Light and electron microscopy demonstrated more severe renal tissue injury in the lumican transgenic mice compared with that in the control mice. In conclusion, LPS may cause excessive apoptosis in the renal tubular cells via the TLR4 signal transduction pathway, a decrease in the number of renal tubular cells and ARF. Lumican may be important in mice with LPS-induced ARF.

## Introduction

Lumican is one of the major extracellular proteins in the interstitial extracellular matrix (ECM) of the skin, corneal stroma, sclera, aorta, muscle, lung, kidney, bone, cartilage and intervertebral discs (1). It is a member of the family of small, leucine-rich proteoglycans (SLRP), with a core protein of 30–50 kDa comprising a signal peptide, a negatively charged N-terminal domain, a highly conserved leucine-rich internal domain and a carboxyl-terminal domain (2). The protein core and the glycan chains of lumican can interact with various cellular effectors (3), including cytokines, growth factors and cell surface receptors, to modulate cell adhesion, proliferation and migration (4). The primary function of lumican is to produce rigidity of collagen fibers (5). In addition, lumican is important in cellular migration and cell differentiation, and has a paracrine function (6); therefore, lumican is classified as a matrikine. Lumican is also involved in cancer cell proliferation and metastasis (7). It has been reported that lumican-deficient (Lum−/−) mice are defective in immunological responses through the Fas-Fas ligand pathway (8). Lumican has also been demonstrated to regulate the host response to pathogen-associated molecular patterns (9). Therefore, the Lum−/− mice are hypo-responsive to bacterial lipopolysaccharide (LPS) endotoxins and Lum−/− macrophages in cell culture produce lower levels of pro-inflammatory cytokines in response to LPS (10). Lumican facilitates the innate immune response by binding LPS and transferring the LPS signal to toll-like receptor (TLR)4. However, the detailed immunological role of lumican remains to be elucidated.

Gram-negative bacteria-induced sepsis remains the leading cause of acute renal failure (11). One of the mechanisms of sepsis-induced acute renal failure involves the release of bacterial endotoxin into the circulation, which activates interconnected inflammatory cascades in the kidney, ultimately leading to renal injury (12,13). The production of inflammatory mediators is important in the pathophysiology of inflammation in renal injury. The present study determined the role of lumican in LPS-induced systemic inflammation and renal injury using a mouse model of endotoxemia. It was demonstrated that lumican exacerbated LPS-induced inflammation and renal injury in murine models of sepsis. The potential mechanism may be partly through the adjustment of the TLR4-nuclear factor (NF)κB pathway.

## Materials and methods

### Experimental animals

C57 mice were cross-bred with EF1-Lum transgenic mice overexpressing lumican under the control of the EF1 promoter. Following genotyping, the transgenic EF1-Lum mice from one line were used in the present study and were compared with age-matched and strain-matched C57 mice. A total of 20 male C57BL/6 mice and 20 lumican transgenic mice at 8–10 weeks of age, weighing 20–25 g, were obtained from the Experimental Animal Center of China Medical University (Shenyang, China). The mice were housed in rooms maintained at 26°C and a 12-h light/dark cycle for at least one week to acclimatize to the surroundings, with free access to water and standard mouse chow. The study was approved by the ethics committee of the China Medical University (Shenyang, China) and conformed with the guide for the care and use of laboratory animals published by the National Institutes of Health (Bethesda, MA, USA).

### Experimental protocols

The mice were randomly divided into four groups: Wild-type control (WT CTR), wild-type LPS (WT LPS), lumican transgenic control (TG CTR) and transgenic LPS (TG LPS). A mouse model of endotoxemia, with intraperitoneal injection of a dose of LPS (*Escherichia coli* serotype 055: B5; Sigma-Aldrich, St. Louis, MO, USA) was used. Various doses of LPS were used (10, 15 and 20 mg/kg body weight) and the lowest dose was selected since it was sufficient to induce septic shock in wild-type mice. Each mouse in the LPS group was administered 10 mg/kg body weight LPS. In the control group, an equal volume of sterile saline (Beijing Tiantan Biological Products Co., Ltd, Beijing, China) was administered. Blood samples (0.5 ml) were collected from the inferior vena cava of the mice while the mice were under anesthesia with isoflurane (2%; Abbott Laboratories Co., Shanghai, China), at 24 h following LPS injection. The blood samples were immediately centrifuged at 1,000 × g for 20 min and the serum was stored at −80°C. Following the collection of blood, the mice were sacrificed by cervical dislocation and the kidneys were excised. Each of these experimental groups included ten mice.

### Measurement of serum creatinine (SCr), blood urea nitrogen (BUN) and cytokines

Blood was collected for the measurement of serum creatinine and BUN, according to the manufacturer's instructions (Nanjing Jiancheng, Nanjing, China). Selected cytokines were measured by standard sandwich ELISA. Mouse ELISA kits for tumor necrosis factor (TNF)α, interleukin (IL)-6, IL-4 and IL-10 were obtained from R&D Systems (Minneapolis, MN, USA). Recombinant cytokines were used as standard controls. The experimental samples, negative controls and diluted standard markers were added into each well. The absorbance was measured using a Biotek ELx808 absorbance reader (BioTek Instruments, Inc., Winooski, VT, USA) 540 nm and the total protein concentration was measured using a Bradford assay kit (Bio-Rad Laboratories, Hercules, CA, USA).

### Determination of the expression levels of TLR4 and NFκB by western blot analysis

Renal tissue samples were lysed with lysis buffer (Sigma-Aldrich) and the protein concentrations were determined using a protein assay kit (Bio-Rad Laboratories). An equal quantity of protein (50 *µ*g) from tissue homogenates was separated using 10% SDS-PAGE and was subsequently transferred onto nitrocellulose membranes (Bio-Rad Laboratories). Following blocking of the membrane with 5% non-fat milk in Tris-buffered saline (Sigma-Aldrich) at room temperature for 1 h, the membrane was incubated with mouse monoclonal primary antibodies against TLR4 (1:1,000; cat. no. 2246) and phospho-NFκB (1:1,000; cat. no. 3036) both purchased from Cell Signaling Technology, Inc. (Danvers, MA, USA) at 4°C for 12 h. The membranes were incubated with horseradish peroxidase-conjugated secondary antibody (Cell Signaling Technology, Inc.) for 1 h and signals were observed using an enhanced chemiluminescence kit (Amersham Pharmacia, GE Healthcare, Amersham, UK). The membranes were subsequently re-probed with a mouse monoclonal antibody against actin (1:1,000; cat. no. 12262) and a rabbit polyclonal antibody against NFκB (1:1,000; cat. no. 8242) both purchased from Cell Signaling Technology, Inc. at room temperature for 2 h as indicators for equal loading of the samples. Western blotting data were quantified by densitometric analysis using ImageJ version 1.38× (NIH Image software, Bethesda, MA, USA). Values are expressed as relative differences following normalization against the expression levels of actin and NFκB.

### Determination of renal cell apoptosis by terminal deoxynucleotidyl transferase-mediated dUTP nickend labeling (TUNEL)

Apoptotic cells were detected using a TUNEL assay kit (Promega Corporation, Madison, WI, USA). Fresh kidney sections were fixed in 10% buffered formalin and embedded in paraffin, and 4-*µ*m slices were stained using the *in situ* Cell Death Detection kit (Promega Corporation) according to the manufacturer's instructions. Three tissue sections from each sample were randomly selected and 10 microscopic fields per section were assessed by two independent observers. In each field, the nuclei were quantified and the percentage of TUNEL-positive nuclei was determined.

### Observation of pathological changes by light microscopy and electron microscopy

24 h following LPS injection, the animals were sacrificed by cervical dislocation, followed by immediate organ collection for histological analysis. Fresh kidney tissue sections were fixed in 10% buffered formalin (Sigma-Aldrich) and embedded in paraffin, and 4-*µ*m sections were stained with hematoxylin and eosin (Sigma-Aldrich). Samples were assessed using an Olympus CX22 light microscope (Olympus, Tokyo, Japan). For the severity (score: 0–3) of renal cortical vacuolization, peritubular/proximal tubule leukocyte infiltration, the percentage of proximal tubule simplification and proximal tubule hypereosinophilia was assessed by an experienced pathologist, in a blinded manner. The kidneys were perfusion-fixed with 1.25% glutaraldehyde (Sigma-Aldrich) in 0.1 M phosphate buffer (pH 7.4) and were subsequently cut in sagittal and horizontal cross sections for image analysis. Sections (1 *µ*m) were dried overnight at 45°C on gelatin-coated slides (Sigma-Aldrich), stained at 60°C for 2 h in giemsa (Sigma-Aldrich), cooled to room temperature, dehydrated, cleaned in xylene and mounted in permount (Sigma-Aldrich). A JEOL 1011 transmission electron microscope with a Hamamatsu Orca-HR Digital Camera (JEOL, Inc., Peabody, MA, USA) and the Advanced Microscopy Techniques Corp. AMT16000B image capture system (Advanced Microscopy Techniques Corp., Danvers, MA, USA) were used. A pathologist analyzed the samples and determined the levels of injury in a blinded manner.

### Statistical analysis

All values are expressed as the mean ± standard error of the mean. Differences were compared by analysis of variance, followed by Bonferroni correction for post-hoc t-test where appropriate. P<0.05 was considered to indicate a statistically significant difference. All statistical tests were performed using SPSS software 13.0 (SPSS, Inc., Chicago, IL, USA).

## Results

### BUN and creatinine changes

The present study assessed the serum BUN and creatinine levels (Table I). LPS injection caused a significant increase in serum BUN and SCr levels compared with those in WT and TG mice in the control group (P<0.05). However, the increase was attenuated in the WT group (P<0.05).

### Release of inflammatory cytokines into renal tissues

**Figure 1:**
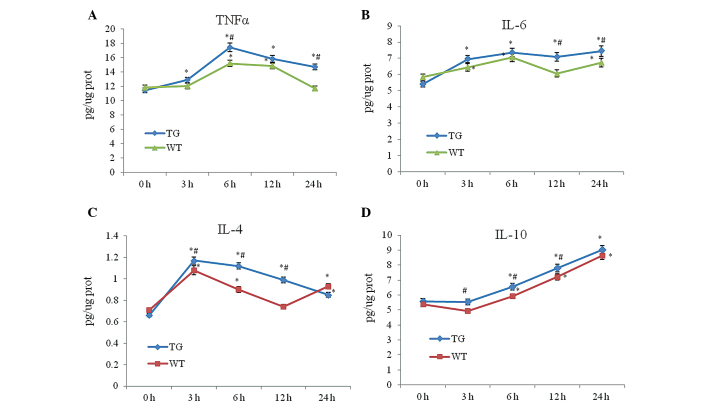
Release of inflammatory cytokines into renal tissue. Following treatment with lipopolysaccharides for 3, 6, 12 and 24 h, renal tissues were collected and selected cytokines, including (A) TNFα, (B) IL-6, (C) IL-4 and (D) IL-10, were measured by standard ELISA. ^*^P<0.05, vs WT CTR; ^#^P<0.05, vs WT LPS. WT, wild-type; TG, transgenic mice; IL, interleukin; TNF, tumor necrosis factor; prot, protein.

LPS treatment increased the expression levels of TNF-α, IL-6, IL-4 and IL-10 compared with those in the control group (P<0.05). However, the expression levels of TNF-α, IL-6, IL-4 and IL-10 in the TG LPS group were significantly higher compared with those in the WT LPS group (P<0.05; Fig. 1).

### Expression levels of TLR4 and NFκB following treatment with LPS

**Figure 2:**
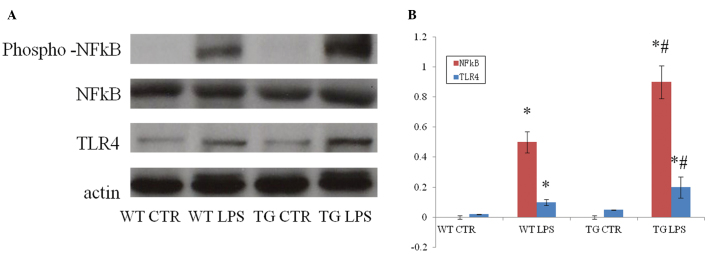
Expression of TLR4 and NFκB following treatment with LPS. (A) Western blot bands of expression levels of TLR4 and NFκB phosphorylation following treatment with LPS. (B) Densitometric values normalized by actin and NFκB. Results are expressed as the mean ± standard error (n=6 for each group). ^*^P<0.05, vs WT CTR; ^#^P<0.05, vs WT LPS. WT, wild type; TG, transgenic mice; CTR, control; LPS, lipopolysaccharide; NF, nuclear factor; TLR, Toll-like receptor.

As shown in Fig. 2, treatment with LPS caused an increase in the expression levels of phospho-NFκB and TLR4 compared with those in the control group in each strain of mice; however, the expression levels of phospho-NFκB and TLR4 were reduced in the WT LPS group compared with those in the TG LPS group.

### Apoptosis in renal tissues following treatment with LPS

**Figure 3:**
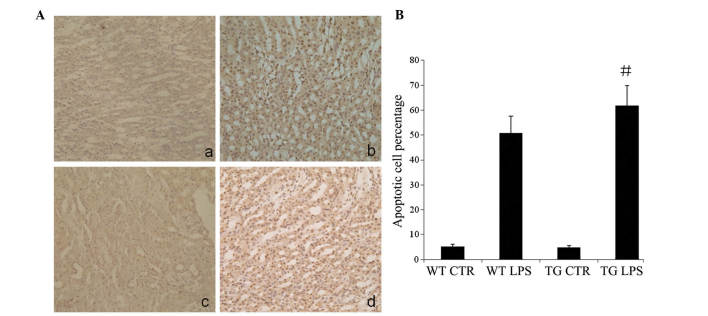
Apoptosis in renal tissues following treatment with LPS. TUNEL staining demonstrated tissue apoptosis following treatment with LPS (magnification, ×200). The percentage of apoptotic nuclei and the total nuclei are expressed as the mean ± standard error of the mean (n=6 for each group). (A) Representative images of TUNEL staining for the various groups. (a) WT CTR group; (b) WT LPS group; (c) TG CTR group; (d) TG LPS group. (B) Percentage of apoptotic cells following various treatments (^#^P<0.05; TG LPS, vs. WT LPS). WT, wild-type mice; TG, transgenic mice; CTR, control; LPS, lipopolysaccharide; TUNEL, Terminal deoxynucleotidyl transferase dUTP nick end labeling.

LPS increased the TUNEL-positive staining in the WT and TG groups compared with that in the control group (P<0.05), particularly in the renal tubular epithelial cells. However, the increase was significantly lower compared with that in the TG LPS group (P<0.05; Fig. 3).

### Pathological changes of kidney tissues

**Figure 4:**
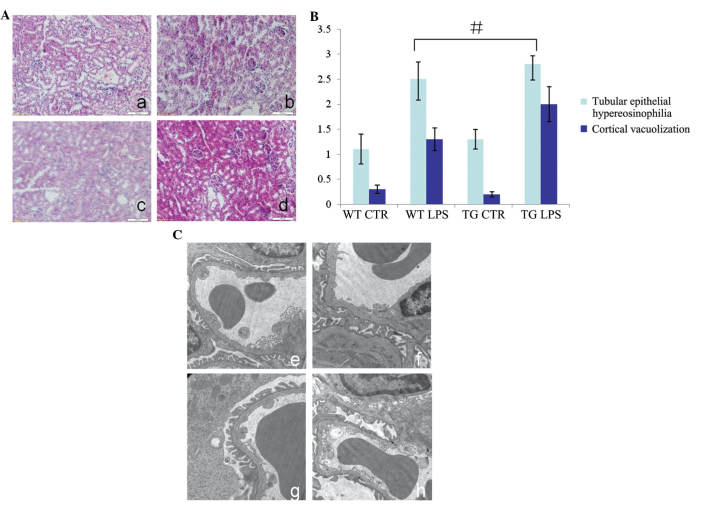
Pathological changes in kidney tissue. Hematoxylin and eosin staining and transmission electron microscopy demonstrating tissue injury following treatment with LPS. (A) Representative images of hematoxylin and eosin staining for the various treatment groups (magnification, ×200). (a) WT CTR group; (b) WT LPS group; (c) TG CTR group; (d) TG LPS group. (B) Injury index of the cells obtained by quantification of A (^#^P<0.05; TG LPS vs. WT LPS). (C) Transmission electron microscopy images for the various groups (magnification, ×25,000). (e) WT CTR group; (f) WT LPS group; (g) TG CTR group; (h) TG LPS group. WT, wild-type mice; TG, transgenic mice; CTR, control; LPS, lipopolysaccharide.

Kidney tissue sections were stained with hematoxylin and eosin, and the samples were assessed for the severity (score: 0–3) of renal cortical vacuolization, peritubular/proximal tubule leukocyte infiltration, percentage of proximal tubule simplification and proximal tubule hypereosinophilia by an experienced pathologist, in a blinded manner. As shown in Fig. 4, the control group exhibited no clear abnormalities under the light microscope, with the exception of occasional edema of renal tubular epithelial cells. In the LPS group, swelling and necrosis of proximal or distal tubules, inflammatory cell infiltration and edema of connective tissue in the renal interstitium was observed (Fig. 4A and B). Administration of LPS also caused glomerular ultrastructural changes in the mice. Transmission electron microscopy of glomeruli from mice which had received LPS demonstrated significant ultrastructural changes compared with the controls (Fig. 4C). None of these pathologic changes were observed in the kidneys of the control mice, whereas in the LPS group, podocytes appeared swollen, foot process effacement was present, the urinary space was diminished and certain samples contained extensive vacuoles of unknown significance.

## Discussion

Proteoglycans are widely distributed in the stromal tissue of mammals and are considered to be closely associated with the ECM and growth factors (14). Proteoglycans belong to the SLRP family and are characterized by the presence of multiple adjacent leucine-rich regions, comprising 20–29 amino acid residues, which may be repeated up to 30 times (2). Members of SLRP family include keratocan, mimecan, decorin, biglycan fibromodulin, epiphycan, osteoadherin and lumican (15). SLRPs, including lumican, have important functions in cell migration, cell proliferation, tissue repair and tumor growth, in addition to their ECM functions in tissue hydration and collagen fibrillogenesis (2). Lumican was first isolated from chick cornea and has also been reported to be localized in the skin dermis, lung, bone, cartilage and heart of adult mice (3). In adult cornea, lumican is present as keratan sulfate proteoglycans (16); however, in non-corneal tissues, lumican exists as low- or non-sulfated glycoprotein (17). Gene-targeting studies indicate that lumican is important in determining the structural phenotype of the mature collagen fibril in various tissue types. In addition, it is hypothesized that lumican sequesters in the pericellular matrix and interacts with cell surface proteins for specific cellular functions. Lumican-deficient mice suffer from increased skin fragility and corneal opacities as a result of abnormal fibril assembly and altered interfibrillar spacing, indicating lateral fusion of collagen fibrils (18). By contrast, lumican mediates Fas-Fas ligand (FasL)-induced apoptosis by inducing Fas in mouse embryonic fibroblasts (19). Lumican, by binding and signaling via FasL, increases the synthesis and secretion of pro-inflammatory cytokines and the recruitment of macrophages and neutrophils (20). The present study demonstrated that bacterial LPS is sensed by the TLR4 signaling pathway and is regulated by lumican. LPS endotoxins from the cell wall of Gram-negative bacteria are recognized by the TLR4 signaling pathway (21). LPS sensing begins with its binding to the LPS-binding protein in the blood. The LPS recognition complex also requires soluble MD-2 protein, heat shock proteins and additional factors, which remain to be identified (22–24). The TLR4 signaling pathway involves the phosphorylation of inhibitor of NF-κB kinase, nuclear translocation and the activation of NF-κB. The NF-κB transcription factor upregulates pro-inflammatory cytokines, including TNFα and IL-6, and increases microbicidal activities (25,26). TNFα is a cytokine prototype and is often used to assess the host innate immune response. These mediators assist in clearing infections. However, unrestricted systemic overproduction of pro-inflammatory cytokines and proteins can lead to severe sepsis, multiple organ failure and mortality. Host response to infection and bacterial endotoxins is being investigated at multiple levels to define the events leading to sepsis and septic shock. Understanding the molecular events from pathogen recognition to inflammatory mediators is becoming important in the treatment of sepsis and for identifying patients at risk.

Bacterial infection causes shock, acute respiratory failure, multiple organ dysfunction syndrome and disseminated intravascular coagulation. Despite an improved understanding of the epidemiology, pathophysiology and genetic pre-disposition to sepsis, morbidity and mortality associated with severe sepsis and septic shock remain high throughout the world (27,28). Renal failure is one of the most common and life-threatening diseases during septic shock.

The present study of the ECM protein identified lumican as a novel modulator of the host response to inflammation and sepsis. Lumican modulates host sensing of bacterial LPS by TLR4 in a mouse model of LPS-induced systemic inflammation. The serum levels of TNF-α, IL-6, IL-4 and IL-10 were examined. These cytokines are normally markedly induced by LPS and are secreted during the early phase of the inflammatory response, therefore exerting an important role in organ dysfunction (29). TNFα is a primary mediator of inflammation and its release leads to the activation of other cytokines, including IL-6, which is associated with cellular damage (30,31). On the other hand, IL-6 may be a more consistent predictor of sepsis and appears to correlate better with sepsis severity and mortality (32). It was demonstrated that Lum+/+ mice are hyper-responsive to LPS-induced septic shock, with further induction of pro-inflammatory cytokines, including TNFα and IL-6, and anti-inflammatory cytokines, including IL-4 and IL-10, in the serum. LPS treatment successfully induced multiple organ dysfunction syndrome in mice, including acute renal failure indicated by increased BUN and SCr levels, increased TUNEL-positive staining, particularly in tubular epithelial cells, and histological damage in the kidney, accompanied with activation of the TLR4 pathway. Lum+/+ mice exhibited a more severe injury compared with wild-type mice.

In conclusion, the present study demonstrated that LPS caused excessive apoptosis of renal tubular cells via the TLR4 signal transduction pathway, decreased the number of renal tubular cells and resulted in ARF. Lumican may be involved in LPS-induced ARF in mice.

## Figures and Tables

**Table I:** Contents of serum BUN and SCr in each group.

Group	BUN (mmol/l)	SCr (*µ*mol/l)
WT CTR	17.3±1.9	49.8±2.3
WT LPS	20.2±1.7^a^	55.9±2.8^a^
TG CTR	18.7±2.3	50.1±2.7
TG LPS	23.7±1.8^a^,^b^	58.2±2.9^a^,^b^

a P<0.05, vs. WT CTR;

b P<0.05, vs. WT LPS. Values are expressed as the mean ± standard error (n=10). WT, wild-type; TG, transgenic mice; CTR, control group; LPS, LPS treatment group; BUN, blood urea nitrogen; SCr, serum creatinine.

## References

[1] Chen S, Birk DE (2013). The regulatory roles of small leucine-rich proteoglycans in extracellular matrix assembly. FEBS J.

[2] Schaefer L (2011). Small leucine-rich proteoglycans in kidney disease. J Am Soc Nephrol.

[3] Nikitovic D, Katonis P, Tsatsakis A, Karamanos NK, Tzanakakis GN (2008). Lumican, a small leucine-rich proteoglycan. IUBMB Life.

[4] Naito Z (2005). Role of the small leucine-rich proteoglycan (SLRP) family in pathological lesions and cancer cell growth. J Nippon Med Sch.

[5] Brézillon S, Pietraszek K, Maquart FX, Wegrowski Y (2013). Lumican effects in the control of tumour progression and their links with metalloproteinases and integrins. FEBS J.

[6] Malinowski M, Pietraszek K, Perreau C (2012). Effect of lumican on the migration of human mesenchymal stem cells and endothelial progenitor cells: involvement of matrix metallopro-teinase-14. PLoS One.

[7] Goldoni S, Iozzo RV (2008). Tumor microenvironment: Modulation by decorin and related molecules harboring leucine-rich tandem motifs. Int J Cancer.

[8] Vij N, Roberts L, Joyce S, Chakravarti S (2005). Lumican regulates corneal inflammatory responses by modulating Fas-Fas ligand signaling. Invest Ophthalmol Vis Sci.

[9] Lohr K, Sardana H, Lee S, Wu F, Huso DL, Hamad AR, Chakravarti S (2012). Extracellular matrix protein lumican regulates inflammation in a mouse model of colitis. Inflamm Bowel Dis.

[10] Shao H, Scott SG, Nakata C, Hamad AR, Chakravarti S (2013). Extracellular matrix protein lumican promotes clearance and resolution of Pseudomonas aeruginosa keratitis in a mouse model. PLoS One.

[11] Howell GM, Gomez H, Collage RD (2013). Augmenting autophagy to treat acute kidney injury during endotoxemia in mice. PLoS One.

[12] Cui WY, Tian AY, Bai T (2011). Protective effects of propofol on endotoxemia-induced acute kidney injury in rats. Clin Exp Pharmacol Physiol.

[13] Nair AR, Masson GS, Ebenezer PJ, Del Piero F, Francis J (2014). Role of TLR4 in lipopolysaccharide-induced acute kidney injury: protection by blueberry. Free Radic Biol Med.

[14] Juneja SC, Veillette C (2013). Defects in tendon, ligament, and enthesis in response to genetic alterations in key proteoglycans and glycoproteins: a review. Arthritis.

[15] Iozzo RV, Schaefer L (2010). Proteoglycans in health and disease: novel regulatory signaling mechanisms evoked by the small leucine-rich proteoglycans. FEBS J.

[16] Amjadi S, Mai K, McCluskey P, Wakefield D (2013). The role of lumican in ocular disease. ISRN Ophthalmol.

[17] Frey H, Schroeder N, Manon-Jensen T, Iozzo RV, Schaefer L (2013). Biological interplay between proteoglycans and their innate immune receptors in inflammation. FEBS J.

[18] Chakravarti S (2002). Functions of lumican and fibromodulin: lessons from knockout mice. Glycoconj J.

[19] Vij N, Roberts L, Joyce S, Chakravarti S (2004). Lumican suppresses cell proliferation and aids Fas-Fas ligand mediated apoptosis: implications in the cornea. Exp Eye Res.

[20] Vij N, Roberts L, Joyce S, Chakravarti S (2005). Lumican regulates corneal inflammatory responses by modulating Fas-Fas ligand signaling. Invest Ophthalmol Vis Sci.

[21] Ramachandran G (2014). Gram-positive and gram-negative bacterial toxins in sepsis: a brief review. Virulence.

[22] Maeshima N, Fernandez RC (2013). Recognition of lipid A variants by the TLR4-MD-2 receptor complex. Front Cell Infect Microbiol.

[23] Tamura Y, Torigoe T, Kutomi G, Hirata K, Sato N (2012). New paradigm for intrinsic function of heat shock proteins as endogenous ligands in inflammation and innate immunity. Curr Mol Med.

[24] Lucas K, Maes M (2013). Role of the Toll Like receptor (TLR) radical cycle in chronic inflammation: possible treatments targeting the TLR4 pathway. Mol Neurobiol.

[25] Hildebrand D, Sahr A, Wölfle SJ, Heeg K, Kubatzky KF (2012). Regulation of Toll-like receptor 4-mediated immune responses through Pasteurella multocida toxin-induced G protein signalling. Cell Commun Signal.

[26] da Silveira Cruz-Machado S, Carvalho-Sousa CE, Tamura EK (2010). TLR4 and CD14 receptors expressed in rat pineal gland trigger NFKB pathway. J Pineal Res.

[27] Schorr CA, Zanotti S, Dellinger RP (2014). Severe sepsis and septic shock: management and performance improvement. Virulence.

[28] Balk RA (2014). Systemic inflammatory response syndrome (SIRS): where did it come from and is it still relevant today?. Virulence.

[29] Li XJ, Zhang GX, Sun N, Sun Y, Yang LZ, Du YJ (2013). Protective effects of erythropoietin on endotoxin-related organ injury in rats. J Huazhong Univ Sci Technolog Med Sci.

[30] Striz I, Brabcova E, Kolesar L, Sekerkova A (2014). Cytokine networking of innate immunity cells: a potential target of therapy. Clin Sci (Lond).

[31] Khosravi R, Ka K, Huang T, Khalili S, Nguyen BH, Nicolau B, Tran SD (2013). Tumor necrosis factor-α and interleukin-6: potential interorgan inflammatory mediators contributing to destructive periodontal disease in obesity or metabolic syndrome. Mediators Inflamm.

[32] Zhao Y, Li C (2014). (Diagnostic value of a combination of biomarkers in patients with sepsis and severe sepsis in emergency department). Chin Crit Care Med.

